# The impact of square dancing on psychological well-being and life satisfaction among aging women

**DOI:** 10.1038/s41598-024-61143-x

**Published:** 2024-05-06

**Authors:** Xi Liu, Qinjin Du, Hongying Fan, Yan Wang

**Affiliations:** 1 School of Art, Beijing Sport University, Beijing, 100084 China; 2 School of Psychology, Beijing Sport University, Beijing, 100084 China; 3 Key Laboratory of Exercise and Physical Fitness (Beijing Sport University), Ministry of Education, Beijing, China; 4 Laboratory of Sports Stress and Adaptation of General Administration of Sport, Beijing, China

**Keywords:** Psychology, Health care

## Abstract

As the most popular sport among middle-aged and elderly women in China, square dancing has both physical and psychological benefits for menopausal women. Previous studies have shown that square dance exercises can promote the physical health of older women, but there is a lack of research on the influence of middle-aged and elderly women on mental health and mediating variables. Therefore, this study starts with one of the important indicators of mental health—positive affects, aiming to explore the impact of square dance on the positive emotions of elderly women and further explore the mediating mechanisms involved. We send out The Physical Activity Rating Scale, the Positive and Negative Affect Scale, the Connor-Davidson Resilience Scale, and the Satisfaction With Life Scale to a total of 2311 middle-aged and elderly women. SPSS 23 software and PROCESS were used to perform regression analysis and establish mediation models. Modeling results show square dance exercises could positively predict positive affect through the chain mediating effect of psychological resilience and life satisfaction. The results of this study are of great significance for promoting the extensive participation of middle-aged and elderly women in sports and protecting their mental health.

## Introduction

The increase in global life expectancy highlights the importance of improving the well-being of middle-aged and older people^1^, especially women who experience the "empty nest phenomenon in midlife"^2^. This phenomenon is prevalent among Chinese urban families, especially those with only one child, and also happens throughout the world gradually, leading to increased depression, loneliness, and other mental issues^3^. The psychological problems mentioned earlier directly affect the quality of life and mental health of middle-aged and elderly women^4^. These conditions not only reduce individual well-being but also have wider social impacts, including increased healthcare costs and reduced social cohesion^5^. Consequently, mental health issues among this group have become a significant concern in the field of geriatric psychology over the past few decades^6^.

This study aims to explore an innovative solution to this problem through the lens of positive psychology. According to Lyubomirsky et al. (Pursuing Happiness: The Architecture of Sustainable Change), engaging in intentional activities can foster sustainable happiness. For instance, incorporating an exercise routine can be highly beneficial^7^. It is important, however, for these intentional activities to remain fresh, meaningful, and positive for individuals to prevent adaptation and sustain their efficacy^8^. Based on this, Physical activity can be a tool and contributes significantly to promoting mental health in elderly adults^9^. Square dance is a popular, moderate-intensity sport that combines physical activity with social interaction and cultural expression^10^, and has been rapidly gaining popularity among middle-aged and elderly groups^11^. Participation in square dancing can effectively improve middle-aged and older adults' self-efficacy, social support, and positive psychological qualities^12^, making it probably an ideal intervention to address the negative impacts of the empty nest phenomenon.

### Chinese square dance exercise and positive affect

Square dance, a rhythmic fitness dance performed spontaneously in communal spaces^13^, is accompanied by high-decibel, rhythmic music and incorporates various dance elements, including traditional Chinese-style dancing, folk dance, modern dance, street dance, and Latin dance. Unlike other forms of exercise, square dancing is community-oriented, easy to participate in, and requires no special equipment or facilities, making it a sustainable option for regular physical activity. In recent years, Chinese seniors have significantly increased their use of parks, and, of all park sports, square dancing has been the fastest-growing fitness sport in recent years and is one of the most popular sports among people over 50^14^. Many studies have shown that participation in square dancing can increase confidence and a sense of accomplishment, relieve stress, and increase a sense of belonging^15^. In addition, previous studies from our research group illustrated that square dance to a certain extent meets the physiological and psychological needs of women, and significantly enhances the happiness index of women^16^. They also verified that square dance can positively predict group cohesion through social support and psychological capital^17^. Therefore, it is most appropriate to choose square dance as the main variable of this study.

Positive affect, conventionally associated with health and happiness^18^, is viewed through Fredrickson's broaden-and-build theory, suggesting that positive emotions contribute to well-being, health, social integration, knowledge, and productivity^19^. Positive emotions can be cultivated to overcome negativity, enhancing psychological well-being and physical health^5^. Some models demonstrate that positive emotions can be effective in combating stress, and it is noted that including positive emotions in research can help address the imbalance between research and clinical practice that has resulted from focusing only on negative emotions in past decades^20^. That's one of the reasons we focus on positive affect. Square dancing's impact is evident in increased confidence, sense of accomplishment, stress relief, and a heightened sense of belonging^15^. Notably, a square dance program has shown cognitive improvement, enhanced quality of life, and reduced depressive symptoms in older Chinese women with mild cognitive impairment^12^. Based on these findings, hypothesis 1 is proposed: Square dance exercise positively correlates with positive affect.

### Psychological resilience and positive affect

Psychological resilience, defined by Block as the ability to adapt and recover from stress^21^, is functionally akin to hardiness^22^. It serves as a crucial mediating variable between physical activity and subjective well-being^23^. Higher psychological resilience in older adults is associated with lower psychological problems^24^, reduced depression risk, enhanced coping with aging, and improved quality of life and lifestyle^25–27^. Hence, this study proposes hypothesis 2: Psychological resilience mediates the relationship between square dance exercise and positive affect.

### Life satisfaction and positive affect

Life satisfaction, a vital well-being indicator, correlates significantly with positive affect^28^. It is a component of subjective well-being and reflects positive mental health^29^. The study classified the physical activities in which the elderly group participated into high, medium, and low categories, and the results showed that participants with high and medium activity levels and their high life satisfaction and happiness were significantly higher than those with low activity levels^30^. Participation in physical activities, such as tai chi and square dance, enhances life satisfaction and happiness in the elderly group^31^. Involvement in sports and dance is linked to increased life satisfaction, with more frequent participation yielding higher satisfaction levels^32,33^. Physical activity and leisure activities negatively predict higher life satisfaction and well-being in older adults^33^. Thus, this study proposes hypothesis 3: Life satisfaction mediates the relationship between square dance exercise and positive affect.

### The chain-mediating effect of psychological resilience and life satisfaction

Elastic development mechanisms, identified in a study by Rishworth and Elliott, include risk impact reduction, psychological resilience, protective mechanisms, negative chain reaction reduction, and self-esteem and self-efficacy promotion^34^. The study infers that square dance, as an intervention, effectively serves as protection and acceptance during encounters with the empty nest and retirement loss, linking psychological support to increased happiness and mitigating negative influences. Research indicates a positive correlation between psychological resilience and life satisfaction, where higher psychological resilience levels are associated with greater life satisfaction^35–37^. A survey with 310 middle-aged adults shows a positive correlation between self-efficacy, psychological resilience, and life satisfaction^38^. Hence, this study proposes hypothesis 4: Psychological resilience and life satisfaction play a chain mediating role in the relationship between square dance exercise and positive affect.

**Figure 1:**
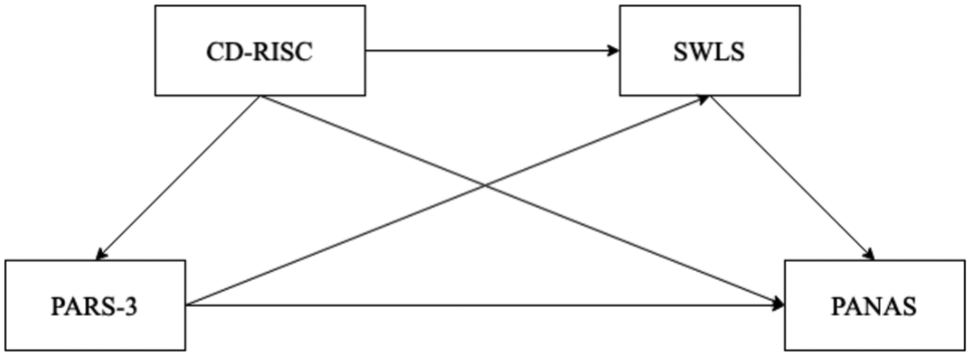
Chain mediating effect model.

Overall, the hypothesized model for this study is shown in Fig. 1, illustrating the interrelationships between the variables.

## Methods

### Participants

The data was collected through a questionnaire survey from April 10th to May 10th, 2023 (https://www.wjx.cn/) Network survey collection was conducted. We have controlled the IP addresses of the respondents to avoid the same person answering multiple times. The collected IP addresses are distributed in 32 provinces, autonomous regions, and municipalities directly under the central government, including Beijing, Jiangsu, Guangdong, Fujian, Inner Mongolia, and Shanghai (sorted by the number of responses received). The initial dataset included 2721 respondents. Our research is in line with the newly revised ethical guidelines of the Declaration of Helsinki.

**Figure 2:**
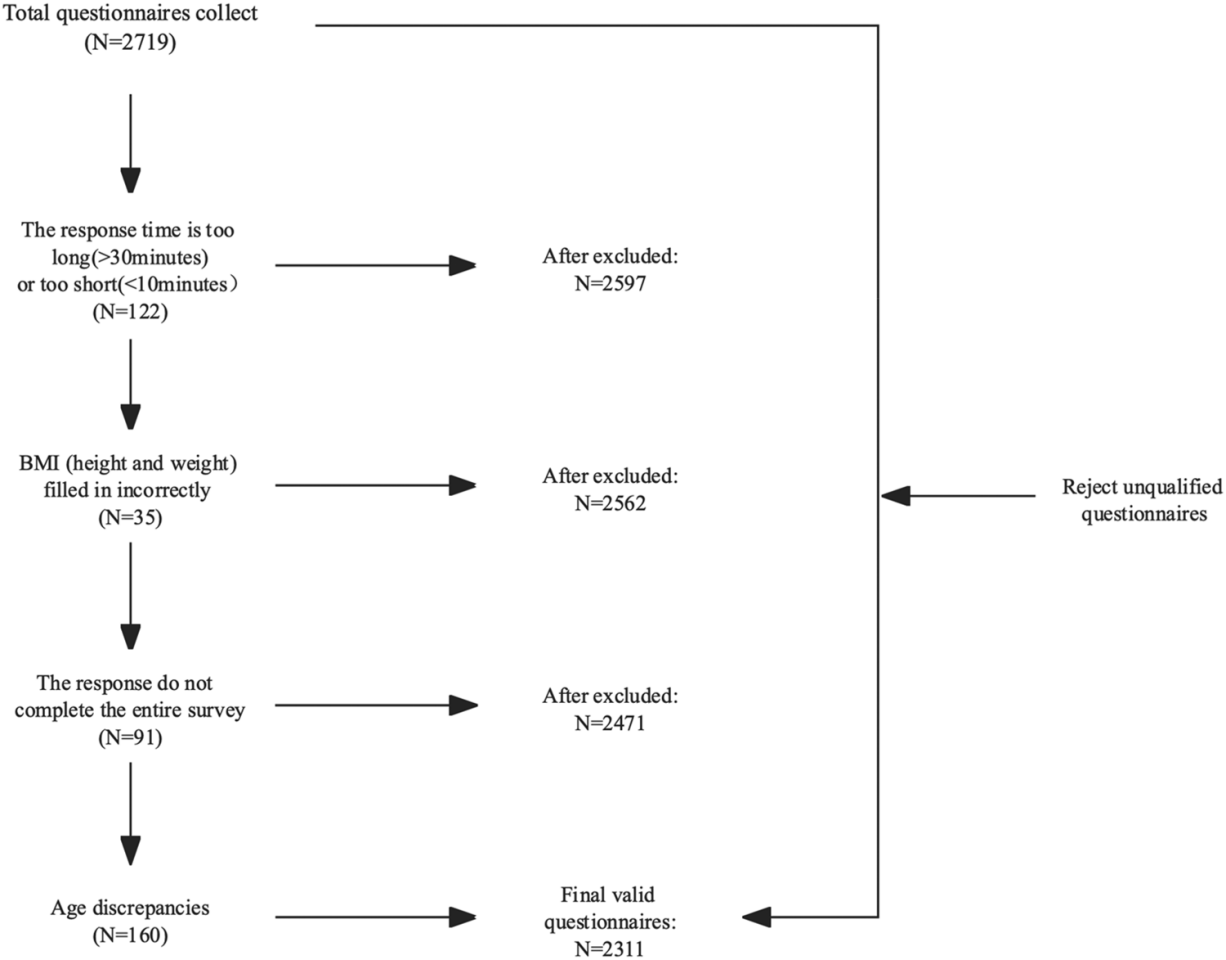
The exclusion process of participants.

The reasons and process for excluding invalid questionnaires are shown in Fig. 2.

We have received questionnaires from all over the country. According to the identification of position, the proportion of participants in the region is as follows (Table 1), and the age of participants is shown in Table 2.

**Table 1:** The basic information of participants (N = 2311).

Sex	Female (100%)
BMI	23.16 ± 3.03
Monthly income	
< 3500	1175 (50.8%)
3500–6500	896 (38.8%)
> 6500	240 (10.4%)
Living condition	
Live alone	107 (4.6%)
Live with others	2204 (95.4%)
Education background	
Compulsory education and below (including primary and junior high schools)	592 (25.6%)
High school (including technical secondary school)	1060 (45.9%)
Undergraduate (including junior college)	640 (27.7%)
Master degree and above	19 (0.8%)
IP address	
East China	749 (32.4%)
North China	679 (29.4%)
South China	528 (22.8%)
Northwest China	137 (5.9%)
Southwest China	115 (5.0%)
Central China	88 (3.8%)
Northeast China	10 (0.4%)
Else regions	5 (0.2%)

**Table 2:** Participants’ age group.

Age/year	45–49	50–54	55–59	60–64	65–69	70–74	Over 75	Total
Population	120	304	577	577	526	178	29	2311
Percentage/%	5.2	13.1	25.0	25.0	22.8	7.7	1.2	100

### Measures

#### Physical Activity Rating Scale (PARS-3)

The Chinese version of the Physical Activity Rating Scale was translated and revised by Liang^63^. The scale consists of three questions, including intensity, duration, and frequency of participating in physical activity. Each question is quantified using a 5-point Likert scale, and each item is rated on a scale of 1–5. Physical activity score = physical activity intensity score × (physical activity time score − 1) × physical activity frequency score, with a score range of 0–100. Based on the overall activity score, physical activity levels were categorized as low, moderate, or high: low intensity ≤ 19 points, moderate intensity ≤ 20–42 points, and high intensity ≤ 43 points.

#### Positive and Negative Affect Scale (PANAS)

The Chinese version of the Positive and Negative Affect Scale was revised by Huang et al.^39^. The questionnaire consists of two dimensions, positive and negative, with a total of 20 items and a 5-point scale. The PA scale (α = 0.85) consists of 10 adjectives describing positive moods. A high score on the PA scale indicates an energetic, attentive, and happy emotional state, while a low score indicates indifference. The NA scale (α = 0.83) consists of 10 adjectives describing negative moods, with a high NA score indicating a subjective feeling of confusion and distress, and a low score indicating calmness. The PANAS has good reliability and validity, with a Cronbach's alpha coefficient of 0.82 for all items. In this experiment, only the PA part is used, and in this study Cronbach's alpha coefficient = 0.92, McDonald's ω = 0.935.

#### Connor-Davidson Resilience Scale (CD-RISC)

The Chinese version of the Connor-Davidson Resilience Scale used in this study was translated and revised by Yu and Zhang^40^. The CD-RISC is based on a study of PTSD, consisting of five factors: ability, tolerance of negative affect, acceptance of change, control, and psychological impact, with a total of 25 items. Since the scale presents different structural models when used in different countries, ages, and life circumstances, the exploratory factor analysis on the Chinese general population resulted in a three-factor model, namely, resilience, strength, and optimism, with high reliability (α = 0. 91). The scale is scored on a 5-point Likert with a total score of 0 to 100, with higher scores indicating greater resilience. In this experiment Cronbach's alpha coefficient = 0.96, McDonald's ω = 0.965.

#### Satisfaction With Life Scale (SWLS)

The Chinese version of the Satisfaction With Life Scale was translated and adapted by Xu and Xiong^65^ and contains five items. Participants indicated their level of agreement on a 5-point Likert scale ranging from 1 (strongly disagree) to 5 (strongly agree), with higher scores indicating greater satisfaction with life. Moreover, this scale correlates as well with other measures of subjective well-being and is suitable for use with different age groups^66^. In the present study, Cronbach’s = 0.79.

Additionally, to reduce potential bias, all questionnaires are given with uniform objective guidance and an emphasis on honest responses before each part of the questionnaire. The following measures are also used: we adopted anonymous surveys to enhance respondents' awareness of privacy protection, reduce their psychological burden, and make it easier to get truthful and objective answers; Choose a relatively objective translation that has been widely used many times to avoid guidance and subjective interference, make the options as clear and specific as possible, and avoid ambiguity and ambiguity; After obtaining the answer data, we repeatedly verified and checked the data to correct the error caused by the answer bias and other reasons.

### Data processing

IBM SPSS Statistics 23.0 and PROCESS macro for SPSS (version 3.2) compiled by Hayes^64^ were used to analyze the data obtained from this questionnaire. To ensure the validity of the data, Harman’s single-factor test of principal component analysis is used to test for bias in commonly used methods. The analysis results show that the first factor is 40.1%, indicating no significant bias in the general method. This proves that the difference between the independent and dependent variables is mainly caused by the characteristics of the variables themselves, rather than by changes in data collection and measurement techniques. After evaluating the bias of commonly used methods, descriptive statistical analysis was conducted using SPSS to calculate the mean and standard deviation of the data. Similarly, SPSS is used to calculate the Pearson correlation coefficients between variables, to examine the degree of interrelationships and patterns of change between independent variables, intermediate variables, and dependent variables; the single-sample t-test was used to examine the differences in the respective variables between women participating in square dancing and unrestricted square dancing groups. Finally, PROCESS macro for SPSS was used for mediation analysis to explore the mediating role of psychological resilience and life satisfaction, and to validate the four hypotheses of this study.

### Common method bias test

To control for common method bias, the questionnaire was distributed anonymously during the survey, and Harman's one-way analysis of variance was used to test the severity of homoscedasticity error in the data of this study. Before factor rotation, the first factor explained 40.1% of the variance, indicating that there was no serious common method bias.

### Institutional review board statement

All methods were carried out according to relevant guidelines and regulations. The studies involving human participants were reviewed and approved by a local Ethics Committee (Ethical Approval Document No. 2023128H). All subjects and/or their legal guardian(s) were provided informed consent to this questionnaire.

## Results

### T-test

We conducted a single sample t-test using IBM SPSS Statistics 23.0 to examine the differences in related factors between women participating in square dancing and other populations. Data analysis shows that the life satisfaction of middle-aged and elderly women participating in square dancing (M = 28.68, SD = 5.36) is significantly higher than that of the middle-aged and elderly population without limited participation in square dancing (M = 18.43, SD = 3.11), t = 91.954, p < 0.001; The psychological resilience of middle-aged and elderly women who participated in square dancing (M = 71.35, SD = 18.93) was significantly higher than that of the unrestricted group of middle-aged and elderly people who participated in square dancing (M = 66.07, SD = 18.24), t = 13.414, p < 0.001; The positive affection (M = 37.00, SD = 7.13) of middle-aged and elderly women participating in square dancing was significantly higher than that of the unrestricted group of middle-aged and elderly people with good overall health and participation in square dancing (M = 30.89, SD = 6.16), t = 41.23, p < 0.001.

Based on the above comparison results, it is proven that square dancing has a positive impact on the life satisfaction, psychological resilience, and positive affection of middle-aged and elderly women.

### Correlation of variables

Correlation analysis was conducted on the four research variables of square dancing exercise amount, psychological resilience, life satisfaction, and positive affect, and the results are shown in Table 3.

**Table 3:** Descriptive statistics and correlation coefficient matrix of each variable.

Variables	M	SD	1	2	3	4	5	6
1. PARS-3	13.84	11.93	–					
2. CD-RISC-tenacity	36.52	10.63	0.088***	–				
3. CD-RISC-strength	24.17	6.00	0.114***	0.885***	–			
4.CD-RISC-optimism	10.66	3.40	0.089***	0.749***	0.758***	–		
5. SWLS	28.68	5.36	0.074***	0.491***	0.468***	0.456***	–	
6. PANAS-PA	37.00	7.13	0.144***	0.587***	0.577***	0.450***	0.451***	–

PARS-3: Physical activity rating scale-3; CD-RISC: Connor-Davidson Resilience Scale; SWLS: Satisfaction With Life Scale; PANAS-PA: positive and negative affect scale-positive affect.

***p < 0.001.

It proves that the amount of square dancing exercise, psychological resilience, life satisfaction, and positive emotions are significantly positively correlated. The amount of square dancing exercise was significantly positively correlated with the results of three dimensions of psychological resilience scale, and was significantly positively correlated with life satisfaction. The three dimensions of the mental resilience scale also showed significant positive correlation with positive affect. It provides preliminary support for further testing the hypothesis.

### Square dance exercise influences positive affect: the mediating effect model of psychological resilience and life satisfaction

As shown in Table 4, the SPSS macro program Process compiled by Hayes was used to analyze the mediating effect of psychological resilience and life satisfaction on the relationship between the amount of square dancing exercise and positive affect. Regression analysis showed that the amount of square dancing exercise had a positive and direct prediction effect on mental resilience (β = 0.102, P < 0.001).psychological resilience had a positive and direct effect on life satisfaction (β = 0.503, P < 0.001), but exercise measure had no significant effect on life satisfaction (β = 0.023, P = 0.198). When the amount of square dancing exercise, psychological resilience, and life satisfaction predicted positive emotional experience at the same time, the amount of square dancing exercise, psychological resilience, and life satisfaction all had a significant positive predictive effects on positive affect (β = 0.080, P < 0.001;β = 0.483, P < 0.001; β = 0.201, P < 0.001).

**Table 4:** Regression analysis between variables.

Regression equation		Overall fit index			Regression coefficient significance		95% confidence interval	
Outcome variable	Predictor variable	R	R2	F	β	t	Lower limit	Upper limit
Psychological resilience		0.102	0.010	24.17***			67.939	70.294
	Square dancing exercise				0.102	4.92***	0.097	0.226
Life satisfaction		0.506	0.256	397.21***			17.623	19.124
	Square dancing exercise				0.023	1.29	-0.006	0.026
	Psychological resilience				0.503	27.88***	0.132	0.152
Positive affect		0.624	0.389	489.19***			14.404	16.963
	Square dancing exercise				0.080	4.91***	0.029	0.067
	Psychological resilience				0.483	25.55***	0.168	0.196
	Life satisfaction				0.201	10.63***	0.218	0.316

***p < 0.001.

**Figure 3:**
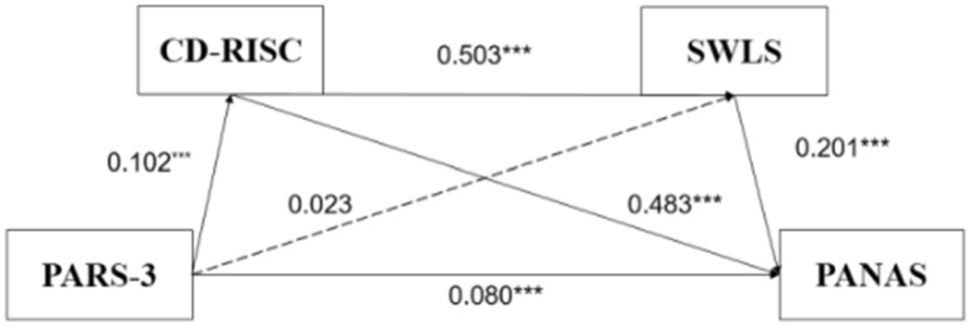
Chain mediating effect model of psychological resilience and life satisfaction between square dance exercise and positive affect. ***p < 0.001. PARS-3: Physical activity rating scale-3, CD-RISC: Connor-Davidson Resilience Scale, SWLS: satisfaction with life scale, PANAS: positive and negative affect scale.

As shown in Table 5, the deviation calibration non-parametric percentile Bootstrap method was used to further test the mediation effect, and the sample size of Bootstrap was set to 5000. The results showed that online positive feedback and positive emotion had a significant mediating effect, and the mediating effect value was 0.038. Specifically, the mediating effect is generated through two mediating chains: First, indirect effect 1 (0.029), which consists of the amount of square dancing exercise → psychological resilience → positive affect. Bootstrap 95% confidence interval does not contain 0, indicating that the mediating effect of online positive feedback is significant. Second, the indirect effect consisted of intensity → life satisfaction → positive affect 2 (0.003), Bootstrap 95% confidence interval contained 0, indicating that life satisfaction had no significant mediating effect on the amount of square dancing and positive emotion experience. Thirdly, indirect effect 3 (0.006) consisted of the square dancing amount of exercise → psychological resilience → life satisfaction → positive affect. Bootstrap 95% confidence interval did not include 0, indicating that psychological resilience and life satisfaction had a significant chain mediating effect between the square dancing amount of exercise and positive effect. The specific path of the effect of the square dance exercise on positive affect is shown in Fig. 3.

**Table 5:** The mediating effect of psychological resilience and life satisfaction on the relationship between square dancing exercise and positive affect.

	Effect	Boot SE	BootLLCI	BootULCI	Effect size
Total indirect effect	0.038	0.008	0.024	0.053	44.2%
Square dancing exercise → psychological resilience → positive affect	0.029	0.006	0.017	0.042	33.7%
Square dancing exercise → life satisfaction → positive affect	0.003	0.002	-0.002	0.007	3.5%
Square dancing exercise → psychological resilience → life satisfaction → positive affect	0.006	0.002	0.003	0.009	7.0%

## Discussion

The results of the study showed that there was a mediating effect of psychological resilience between square dance exercise and positive affect, which proved that hypothesis 1 was valid; the chain mediating effect of psychological resilience and life satisfaction between the amount of square dance exercise and positive affect was significant, which proved that hypothesis 3 was valid; however, the results of this experiment did not show that the mediating effect of life satisfaction between the amount of square dance exercise and positive affect was valid.

A wave of population aging is sweeping the world, with the number of people aged 60 and older expected to more than double by 2050^34^. In this situation, the mental health issues of older adults should receive more attention. The U.S. Federal Interagency Forum on Aging-Related Statistics (2020) represented that the women ages 55–64 reporting depressive symptoms ranged between 16 and 18%. Consequently, the focus on mental health, particularly among middle-aged and elderly women, assumes heightened significance. This study delves into three pivotal variables—psychological resilience, life satisfaction, and positive affect—all of which exhibit robust correlations with the mental health status of older women.

First and foremost, psychological flexibility, as one of the mediating variables in this experiment, was directly influenced by square dance exercises. The current systematic review and meta-analysis show that resilience is a modifiable factor with great protective potential for older adults' health in general and their mental health in particular^41^. What's more, previous studies have consistently found that life satisfaction is associated with several indicators of a good life at the individual (e.g., better physical and mental health and longer life expectancy), organizational (higher performance and job satisfaction), and societal (higher trust and cooperation, and more pro-social behaviors) levels^42^. In addition, a large number of studies have shown that positive affect is associated with better health and longer life expectancy^43^. In conclusion, this experiment's study of these three variables is beneficial in tackling psychological problems and catering to the emotional needs of seniors.

The benefits of square dance may derive from its multifaceted nature, and by combining the benefits of physical exercise, music and art, and social interaction, the dance intervention may also reduce anxiety and depression^15^. Previous research has shown that due to humans' innate connection to nature, reduced exposure to natural environments such as parks and woodlands is associated with decreased mental health and satisfaction^44^. Even brief experiences in nature have been quoted as having restorative qualities, such as reducing stress, promoting positive affect/attitudes, and providing a sense of relief from mental and physical exhaustion^45^. Square dancing, as a group outdoor sport, often performed in parks or around green spaces, can meet older adults' need for contact with nature and refresh them. However, this experiment is only a cross-sectional study of the benefits of square dancing, and the exact mechanisms of influence have yet to be confirmed by further research.

The greatest benefit of square dance is that it is easy to popularize has the value of fitness, heart and brain health^46^, and is closely related to the modern lifestyle, which can play an unexpected fitness role in entertainment. Therefore, it is more suitable for middle-aged and elderly people to participate in square dance than other types of sports, and they are more inclined to choose square dance as their daily exercise.

The results of this study revealed that the direct effect of square dancing and positive affect was significant and positively correlated. This is consistent with previous studies. Square dancing is a group dance activity performed to multiple types of music. Previous studies have shown that music can trigger the release of endorphins^47^, thereby eliciting affective responses such as heightened positive rather than negative mood effects^48^. Not only that, dance as a well-established exercise therapy has also been shown to have significant positive effects on subjective well-being and positive mood^49^. In a study comparing dance, music listening, and cycling in a non-clinical population, both dancing and passive music listening were shown to enhance positive emotions, reduce negative emotions, and decrease fatigue. This finding supports the use of short periods of dancing or passive music listening as potentially powerful tools to enhance emotional well-being and positive affect in non-clinical settings^50^. This is strong evidence that square dance exercise, which integrates both music and dance, has the opportunity to be a powerful tool to enhance positive affect in older adults.

The results of the correlation analysis in this study indicated that the effect of square dance exercise on positive mood worked through three mediators: through the independent mediation of life satisfaction, through the independent mediation of psychological resilience, and the chain mediation of life satisfaction and psychological resilience. Square dance exercise, psychological resilience, life satisfaction, and positive affect were all significantly and positively correlated. These findings provide new perspectives for exploring the mechanisms of the effects of square dance exercises on promoting healthy psychological states.

Psychological resilience mediates between square dance exercise and positive affect and is an important pathway for the effect of square dance exercise on positive affect, validating hypothesis 2. People with high psychological resilience have highly positive emotional experiences^21^ and experience positive affect even under stress. Several studies have demonstrated a positive correlation between psychological resilience and positive emotional experiences^51,52^, which is consistent with the results of the present study and further validates the facilitative effect of square dance exercise on psychological resilience in middle-aged and older individuals.

This study did not test hypothesis 3, that the direct effect between square dance exercise and life satisfaction was not significant, but there was a significant positive correlation between the two, further supporting the results of previous studies^53^. Cruz-Ferreira verified the direct effect between dance and life satisfaction through a 24-week intervention positive correlation^54^. An experiment demonstrated that weekly creative dance practice over eight months increased life satisfaction in women over 65 years of age^55^. The inability of square dance exercise to directly predict life satisfaction may be due to other variables, such as individual personality characteristics, socioeconomic status^56^, family relationships^57^, and health status, which may have a more direct effect on life satisfaction, thus weakening the direct benefits between square dance exercise and life satisfaction.

Square dance exercise can influence positive affect in middle-aged and older adults through a chain mediating effect of psychological resilience and life satisfaction, and hypothesis 4 was tested. This implies that when middle-aged and older women have higher psychological resilience and life satisfaction, they are more likely to experience positive emotions such as joy and optimism. Psychological resilience and life satisfaction can enhance the positive effect of middle-aged and older adults by improving their ability to regulate their emotions and cope with stress. Psychological resilience and life satisfaction were found to be significantly positively correlated and positively predictive, which is consistent with the findings of other scholars^58,59^. A survey of 1395 community women aged 60 years and older concluded that there was a positive relationship between psychological resilience and the well-being and life satisfaction of middle-aged and older ladies and that psychological resilience predicted the well-being and life satisfaction of middle-aged and older women^60^. This chain-mediated mechanism of action explains that square dance exercise increases psychological resilience, the increase in the level of psychological resilience enhances the life satisfaction of middle-aged and elderly people, and the higher life satisfaction contributes to a positive effect.

In addition to these, The range of R2 is 0–1, and the closer it is to 1, the better the fit of the function, and the more it can predict the change of the dependent variable through the fitted function. The corresponding p-value indicates whether the function is significant, in other words, whether the influence of the independent variable on the dependent variable is significant. In this experiment, the p-value < 0.001 but R 2 A lower value indicates that square dancing does have a significant impact on psychological resilience, but it may not be suitable to fit the changes with a function. Given the similarity of movements, we have consulted numerous literature related to dance. Previous literature has shown that dance is a pathway to enhances psychological resilience^61^; Another study of chair-based dance intervention for elderly people in a nursing home in Macau showed that the scores of psychological resilience of elderly people improved significantly after receiving chair dance intervention^62^. This indicates that our research findings are similar to previous studies and have positive implications. The research on Chinese square dance is an emerging field, and there is not yet much previous literature on the relationship between square dance and psychological resilience. We speculate that the possible reason is that when square dancing affects psychological resilience, there are other mediating factors or possible mechanisms that lead to poor predictive performance of the fitting function. These can become new directions for further research and exploration.

However, there are some shortcomings in the study. Firstly, this study is a cross-sectional survey lacking long-term tracking of participants. Although previous theories and empirical results have provided a certain foundation for this study, it is difficult to infer the causal relationship between variables. Future research needs to combine experiments and subsequent studies to collect more data for more in-depth multidimensional analysis and verification, to reveal the exact causal relationship between variables. Secondly, this study is only aimed at middle-aged and elderly women. Whether the research results can be extended to other genders or age groups still requires further intervention and investigation to confirm. Finally, in addition to the indicators included in this study, other sociodemographic variables are included, such as education level, presence of children, income level, history of chronic diseases, and other important psychological variables such as loneliness, anxiety, self-esteem, etc. The effects of these variables have not been ruled out. This is also one of the possible reasons why the direct impact of square dance on life satisfaction is not significant. It can be included in future related research.

## Conclusions

In conclusion, our study confirmed that square dance exercise positively predicted positive affect, that psychological resilience mediated the relationship between square dance exercise and positive affect, and that psychological resilience and life satisfaction played a chain mediating role in the relationship between square dance exercise and positive affect; however, the present experimental data indicated that life satisfaction did not mediate the relationship between square dance exercise and positive affect.

This study revealed the relationship between square dance exercise and positive affect and constructed a chain mediation model, which has important theoretical value for how to effectively improve positive affect in middle-aged and older adults. The results suggest that people can improve life satisfaction and mental resilience through square dancing movements, thereby promoting positive emotional experiences for individuals and providing them with a better lifestyle. Therefore, we also highly recommend that subsequent consideration be given to combining the square dance movement with other psychological interventions to achieve better results. In the future, researchers can further explore the influence of square dancing on the development of various brain regions from the direction of brain neuroscience, and explore the mode of action of the relationship between square dancing and positive emotions from the direction of brain science.

## Data Availability

All data are available within this manuscript.

## References

[1] Hsu M, Liao P, Zhao M (2018). Demographic change and long-term growth in China: Past developments and the future challenge of aging. Rev. Dev. Econ..

[2] Song, Y. *Chinese Square Dance, Media, and Ideological Dynamics in Contemporary China* (2015). Accessed 4 May 2024.

[3] Fahrenberg B (1986). Coping with the empty nest situation as a developmental task for the aging female—an analysis of the literature. Z. Gerontol..

[4] Zhai Y (2015). Association of empty nest with depressive symptom in a Chinese elderly population: A cross-sectional study. J. Affect. Disord..

[5] Fredrickson BL (2001). The role of positive emotions in positive psychology: The broaden-and-build theory of positive emotions. Am. Psychol..

[6] Organization, W. H. *World Report on Ageing and Health* (World Health Organization, 2015).

[7] Lyubomirsky S, Sheldon KM, Schkade D (2005). Pursuing happiness: The architecture of sustainable change. Rev. Gen. Psychol..

[8] McAlister, L. A dynamic attribute satiation model of variety-seeking behavior. *J. Consum. Res.* **9**(2), 141–150 (1982).

[9] Wong MYC, Ou K, Chung PK, Chui KYK, Zhang C (2023). The relationship between physical activity, physical health, and mental health among older Chinese adults: A scoping review. Front. Public Health.

[10] Ou K, Wong MYC, Chung PK, Chui KYK (2022). Effect of square dance interventions on physical and mental health among Chinese older adults: A systematic review. Int. J. Environ. Res. Public Health.

[11] Ji P, Zhou S, Wang R, Fan H, Wang Y (2022). Subjective exercise experience and group cohesion among Chinese participating in square dance: A moderated mediation model of years of participation and gender. Int. J. Environ. Res. Public Health.

[12] Chang J (2021). The effect of Chinese square dance exercise on cognitive function in older women with mild cognitive impairment: The mediating effect of mood status and quality of life. Front. Psychiatry.

[13] Zhu, Y., Zhang, Y., Wang, Y., & Trivic, Z. The Square Dance in China: How Sensory Design Can Foster Inter-Generational Interaction and Improve Older Adults’ Wellbeing. *J. Urban Design Ment. Health* **7**, 7 (2021).

[14] Deng, C., Feng, R. & Kong, L. Square dance the key factor of the elevating prevalence of physical activity in China. *Iran. J. Public Health* **48**(10), 1920–1921 (2020). PMC6908920 31850272

[15] Yao X, Zhao Y, Yin M, Li Z (2021). Acceptability and feasibility of public square dancing for community senior citizens with mild cognitive impairment and depressive symptoms: A pilot study. Int. J. Nurs. Sci..

[16] Sun Y, Ji P, Wang Y, Fan H (2021). The association between the subjective exercise experience of Chinese women participating in square dance and group cohesion: The mediating effect of income. Front. Psychol..

[17] Qu Y, Liu Z, Wang Y, Chang L, Fan H (2023). Relationships among square dance, group cohesion, perceived social support, and psychological capital in 2721 middle-aged and older adults in China. Healthcare.

[18] Diener, E., Suh, E. M., Lucas, R. E., & Smith, H. L. Subjective well-being: Three decades of progress. *Psychol. Bull.* **125**(2), 276 (1999).

[19] Kok, B. E., Catalino, L. I. & Fredrickson, B. L. The broadening, building, buffering effects of positive emotions. In *Positive Psychology: Exploring the Best in People, Vol 2: Capitalizing on Emotional Experiences* 1–19 (Praeger Publishers/Greenwood Publishing Group, 2008).

[20] Folkman S (2008). The case for positive emotions in the stress process. Anxiety, Stress Coping.

[21] Block J, Kremen A (1996). IQ and ego-resiliency: Conceptual and empirical connections and separateness. J. Pers. Soc. Psychol..

[22] Wright MO, Masten AS, Goldstein S, Brooks RB (2005). Resilience processes in development. Handbook of Resilience in Children.

[23] Kukihara H (2018). The mediating effects of resilience, morale, and sense of coherence between physical activity and perceived physical/mental health among Japanese community-dwelling older adults: A cross-sectional study. J. Aging Phys. Activity.

[24] Wermelinger Ávila MP (2022). Resilience and mental health among regularly and intermittently active older adults: Results from a four-year longitudinal study. J. Appl. Gerontol..

[25] Smith JL, Hollinger-Smith L (2015). Savoring, resilience, and psychological well-being in older adults. Aging Ment. Health.

[26] Hildon Z, Montgomery SM, Blane D, Wiggins RD, Netuveli G (2010). Examining resilience of quality of life in the face of health-related and psychosocial adversity at older ages: What is “right” about the way we age?. Gerontologist.

[27] Nygren B (2005). Resilience, sense of coherence, purpose in life and self-transcendence in relation to perceived physical and mental health among the oldest old. Aging Ment. Health.

[28] Bastian B, Kuppens P, De Roover K, Diener E (2014). Is valuing positive emotion associated with life satisfaction?. Emotion.

[29] Steger, M. F., Oishi, S., & Kesebir, S. Is a life without meaning satisfying? The moderating role of the search for meaning in satisfaction with life judgments.* J. Posit. Psychol.* **6**, 173–180 (2011).

[30] An H-Y (2020). The relationships between physical activity and life satisfaction and happiness among young, middle-aged, and older adults. Int. J. Environ. Res. Public Health.

[31] Yao, Y. *Leisure and life satisfaction among Tai Chi and public square dance participants in Hong Kong* (2015). Accessed 4 May 2024. https://commons.ln.edu.hk/soc_etd/38/.

[32] Paupério T, Corte-Real N, Dias C, Fonseca A (2012). Sport, substance use and satisfaction with life: What relationship?. Eur. J. Sport Sci..

[33] Menec V, Chipperfield J (1997). Remaining active in later life: The role of health locus of control in seniors’ activity level, health, and life satisfaction. J. Aging Health.

[34] Rishworth A, Elliott SJ (2019). Global environmental change in an aging world: The role of space, place and scale. Soc. Sci. Med..

[35] Zheng W, Huang Y, Fu Y (2020). Mediating effects of psychological resilience on life satisfaction among older adults: A cross-sectional study in China. Health Soc. Care Community.

[36] Kong F, Wang X, Hu S, Liu J (2015). Neural correlates of psychological resilience and their relation to life satisfaction in a sample of healthy young adults. NeuroImage.

[37] Temiz ZT, Comert IT (2018). The relationship between life satisfaction, attachment styles, and psychological resilience in university students. Dusunen Adam.

[38] Tagay, O., Karatas, Z., Bayar, O., & Savi-Cakar, F. Resilience and life satisfaction as the predictors of general self-efficacy. *Glob. J. Guid. Counsel. Sch. Curr. Pers.* **6**, 11–17 (2016).

[39] Huang L, Yang T, Li Z (2003). Applicability of the positive and negative affect scale in Chinese. Chin. Ment. Health J..

[40] Yu X, Zhang J (2007). Factor analysis and psychometric evaluation of the Connor-Davidson resilience scale (CD-RISC) with Chinese people. Soc. Behav. Pers..

[41] Färber F, Rosendahl J (2020). Trait resilience and mental health in older adults: A meta-analytic review. Pers. Ment. Health.

[42] The Centre for Bhutan Studies and GNH*. Happiness: Transforming the Development Landscape* (Bhutan, 2017).

[43] Diener E, Chan MY (2011). Happy people live longer: subjective well-being contributes to health and longevity: Health benefits of happiness. Appl. Psychol. Health Well-Being.

[44] Bratman GN, Daily GC, Levy BJ, Gross JJ (2015). The benefits of nature experience: Improved affect and cognition. Landsc. Urban Plan..

[45] Yuen HK, Jenkins GR (2020). Factors associated with changes in subjective well-being immediately after urban park visit. Int. J. Environ. Health Res..

[46] Peng F (2020). Exploring factors influencing whether residents participate in square dancing using social cognitive theory: A cross-sectional survey in Chongqing, China. Medicine.

[47] Chiu C-H (2017). Benefits of different intensity of aerobic exercise in modulating body composition among obese young adults: A pilot randomized controlled trial. Health Qual. Life Outcomes.

[48] Dunbar, R. I. M. *Mind the gap: Or why humans aren’t just great apes* (Oxford University Press, 2014).

[49] Koch S, Kunz T, Lykou S, Cruz R (2014). Effects of dance movement therapy and dance on health-related psychological outcomes: A meta-analysis. Arts Psychother..

[50] Campion M, Levita L (2014). Enhancing positive affect and divergent thinking abilities: Play some music and dance. J. Posit. Psychol..

[51] Cohn MA, Fredrickson BL, Brown SL, Mikels JA, Conway AM (2009). Happiness unpacked: Positive emotions increase life satisfaction by building resilience. Emotion.

[52] Onwukwe, Y. U. *The relationship between positive emotions and psychological resilience in persons experiencing traumatic crisis: A quantitative approach* (Doctoral dissertation, Capella University, 2010).

[53] Sani SHZ (2016). Physical activity and self-esteem: Testing direct and indirect relationships associated with psychological and physical mechanisms. NDT.

[54] Cruz-Ferreira A, Marmeleira J, Formigo A, Gomes D, Fernandes J (2015). Creative dance improves physical fitness and life satisfaction in older women. Res. Aging.

[55] Osgood NJ, Meyers BS, Orchowsky S (1990). The impact of creative dance and movement training on the life satisfaction of older adults: An exploratory study. J. Appl .Gerontol..

[56] Boyce C, Wood A, Powdthavee N (2012). Is personality fixed? Personality changes as much as “variable” economic factors and more strongly predicts changes to life satisfaction. Soc. Indic. Res..

[57] Edwards JN, Klemmack DL (1973). Correlates of life satisfaction: A re-examination. J. Gerontol..

[58] Shi M, Wang X, Bian Y, Wang L (2015). The mediating role of resilience in the relationship between stress and life satisfaction among Chinese medical students: A cross-sectional study. BMC Med. Educ..

[59] Kim J (2019). Nursing students’ relationships among resilience, life satisfaction, psychological well-being, and attitude to death. Korean J. Med. Educ..

[60] Lamond AJ (2008). Measurement and predictors of resilience among community-dwelling older women. J. Psychiatr. Res..

[61] Buck R, Snook B (2020). How might creative learning through dance support resilience?. J. Hum. Behav. Soc. Environ..

[62] Ho V, Li X, Smith GD (2022). An exploratory study to assess the impact of a chair-based dance intervention among older people with depressive symptoms in residential care. Top. Geriatr. Rehabil..

[63] Liang, D. Q. Stress level of college students and its relationship with physical exercise. *Chinese J. Ment. Health* **1**, 5–6 (1994), Accessed 4 May 2024 (Chinese).

[64] Hayes, A. F. *Introduction to mediation, moderation, and conditional process analysis.* (Guilford Publications, 2013).

[65] Xu, Y. L. & Xiong, C. Q. The predictive effect of emotional intelligence on subjective well-being of college students. *Psychol. Res.* **4**, 77–81 (2009), Accessed 4 May 2024 (Chinese).

[66] Diener, R. A. The independence of positive and negative affect. *J. Pers. Soc. Psychol.* **47**, 1105–1117 (1985). 10.1037//0022-3514.47.5.1105 6520704

